# Evaluation of the Anti-Inflammatory/Immunomodulatory Effect of *Teucrium montanum* L. Extract in Collagen-Induced Arthritis in Rats

**DOI:** 10.3390/biology13100818

**Published:** 2024-10-12

**Authors:** Biljana Bufan, Mirjana Marčetić, Jasmina Djuretić, Ivana Ćuruvija, Veljko Blagojević, Dragana D. Božić, Violeta Milutinović, Radmila Janković, Jelena Sopta, Jelena Kotur-Stevuljević, Nevena Arsenović-Ranin

**Affiliations:** 1 Department of Microbiology and Immunology, University of Belgrade-Faculty of Pharmacy, 11221 Belgrade, Serbia; bbiljana@pharmacy.bg.ac.rs (B.B.); dragana.bozic@pharmacy.bg.ac.rs (D.D.B.); 2 Department of Pharmacognosy, University of Belgrade-Faculty of Pharmacy, 11221 Belgrade, Serbia; mirjana.marcetic@pharmacy.bg.ac.rs (M.M.); violeta.milutinovic@pharmacy.bg.ac.rs (V.M.); 3 Department of Pathobiology, University of Belgrade-Faculty of Pharmacy, 11221 Belgrade, Serbia; jasmina.djuretic@pharmacy.bg.ac.rs; 4 Institute of Virology, Vaccines and Sera “Torlak”, 11221 Belgrade, Serbia; ivanajakovljev1@gmail.com (I.Ć.); veljko.blagojevic1988@gmail.com (V.B.); 5 Institute of Pathology “Prof. dr Đorđe Joannović”, University of Belgrade-Faculty of Medicine, 11000 Belgrade, Serbia; radmila.jankovic@med.bg.ac.rs (R.J.); jslabic@yahoo.com (J.S.); 6 Department of Medical Biochemistry, University of Belgrade-Faculty of Pharmacy, 11221 Belgrade, Serbia; jelena.kotur@pharmacy.bg.ac.rs

**Keywords:** *Teucrium montanum* L., collagen-induced arthritis, anti-inflammatory effect, immunomodulatory effect, Th17/T regulatory cell balance

## Abstract

**Simple Summary:**

The use of medicinal traditional plants and plant-derived compounds in the treatment of inflammatory states, including rheumatoid arthritis, is gaining attention due to their anti-inflammatory and immunomodulatory properties and fewer side effects compared to conventional drugs. The current study aimed to evaluate the therapeutic effects of the Mediterranean plant *Teucrium montanum* L. on collagen-induced arthritis in rats, in an in vivo animal model of rheumatoid arthritis. The effect of the *Teucrium montanum* extract was evaluated by examining the cellular and molecular components of innate and adaptive immunity that have been shown to play an important role in the pathogenesis of both rheumatoid arthritis and collagen-induced arthritis. The *Teucrium montanum* extract alleviated the clinical manifestation and improved the histopathological findings of arthritis in CIA. The extract improved the anti-/pro-oxidative balance in serum, suppressed the production of pro-inflammatory cytokines in affected joints, and suppressed the T cell responses in secondary lymphoid organs and the production of autoantibodies. Our results suggest the anti-inflammatory/immunomodulatory properties of *Teucrium montanum* extract and represent a basis for the further exploration of the possible therapeutic use of the extract or its compound/s.

**Abstract:**

The anti-inflammatory/immunomodulatory effects of *Teucrium montanum* L. (TM), a plant distributed in the Mediterranean region, have been insufficiently examined. The effects of the TM ethanol extract were tested in a rat collagen-induced arthritis (CIA) model of rheumatoid arthritis. LC-MS was used for the phytochemical analysis of the TM extract. *Dark Agouti* rats were immunized with bovine type II collagen (CII) in incomplete Freund’s adjuvant for CIA, and treated with 100 or 200 mg/kg of TM extract daily via oral administration. Clinical and histopathological evaluations and a flow cytometric analysis of the phenotypic and functional characteristics of splenocytes and draining lymph node cells were performed. The cytokines in the paw tissue culture supernatants and anti-CII antibodies in serum were determined by ELISA. The TM extract, with the dominant components verbascoside and luteolin 7-*O*-rutinoside, reduced the arthritic score and ankle joint inflammation in CIA rats, promoted the antioxidant profile in serum, and lowered pro-inflammatory TNF-α, IL-6 and IL-1β production. It suppressed the activation status of CD11b+ cells by lowering CD86, MHCII and TLR-4 expression, and promoted the Th17/T regulatory cell (Tregs) balance towards Tregs. A lower frequency of B cells was accompanied by a lower level of anti-CII antibodies in treated rats. These findings imply the favorable effect of TM extract on the clinical presentation of CIA, suggesting its anti-inflammatory/immunomodulatory action and potential therapeutic effect.

## 1. Introduction

Rheumatoid arthritis (RA) is a chronic inflammatory autoimmune disease that attacks multiple small synovial joints symmetrically, starting usually in the joints of the hands and feet. This disease is considered systemic since extra-articular organs such as the heart, lungs, blood vessels and eyes can be involved, too [1]. It is estimated that in 2020, 17.6 million people of all ages had this diagnosis, and in 2050, this number is expected to be around 80% more, i.e., 31.7 million people affected [1]. Rheumatoid arthritis is more prevalent in females, with a female-to-male prevalence rate of 2.45, and a peak incidence between 65 and 80 years of age [1]. Although the precise cause is unknown, numerous genetic factors, such as class II major histocompatibility complex (MHC) molecules in particular, and environmental factors (e.g., viral infection, smoking, diet, microbiota composition) have been associated with an increased risk of RA development and an increase in its severity and progression [1,2,3].

It is hypothesized that the pathogenesis of RA starts at the level of mucosa, probably respiratory mucosa, where various processes can influence the development of autoimmunity and a systemic immune reaction [4]. After initial triggering events, the pathogenic process is transferred to the synovial membrane [2]. The resident cells of the synovial membrane, i.e., synovial fibroblasts and macrophages, are critical components in orchestrating the joint microenvironment and the induction of the inflammatory cytokine milieu [2,5]. These cells secret pro-inflammatory cytokines like tumor necrosis factor (TNF)-α, interleukin (IL)-6, IL-8, and matrix metalloproteinases (MMPs), and thus contribute to cartilage and bone degradation, joint destruction and the maintenance of the inflammatory process in the synovium [3]. Furthermore, there is an influx of immune cells into the affected tissue, guided by the increased expression of adhesion molecules and chemokines. These cells, including dendritic cells, T cells and B cells, contribute to the propagation, intensity and development of inflammation. Both RA and the experimental model of RA, collagen-induced arthritis (CIA), are characterized by a robust and sustained T cell response, and the two most important CD4+ T cell effector subsets involved are CD4+ T helper (Th)1 and Th17 cells [6,7]. The activation of these cells results I then production of pro-inflammatory cytokines including interferon (IFN)-γ, TNF-α, IL-6, granulocyte-macrophage colony-stimulating factor (GM-CSF), IL-17 and-IL22, which accelerate the inflammatory responses and eventually lead to bone destruction and cartilage damage [8]. Regulatory T cells (Tregs) have also been found to be important in the outset and progression of RA. Changes in the number and/or function of Tregs and a disturbed Th17/Tregs balance in animal models of RA and human RA have been shown to play a role in the pathogenesis and development of RA [9,10]. Last but not least, activated B cells produce autoantibodies that form immune complexes that are deposited in joints and induce inflammation in the synovium [11], suggesting the importance of B cells in the development and progression of RA.

Non-steroidal anti-inflammatory drugs, synthetic disease-modifying antirheumatic drugs (DMARDs) (methotrexate, hydrochloroquine, sulfadiazine), biological DMARDs (monoclonal antibodies and recombinant fusion proteins) and targeted synthetic DMARDs (Jak inhibitors) are currently used in the treatment of RA [12,13,14]. However, lifelong therapy with synthetic drugs leads to serious side effects, or in the case of biological DMARDs, to the development of resistance and high financial requirements for the health care system [14]. Therefore, new therapeutic agents with low toxicity and high efficacy have been underexplored. In this context, traditional medicinal plants have shown potential in the safe and effective treatment of RA [15,16,17]. They can be used as a complementary therapy to decrease the need for pharmacological agents or as a safer alternative treatment with equal or better efficacy [16]. The anti-arthritic activity of natural plant compounds, such as flavonoids (e.g., Apigenin, Quercetin, Genistein), phenolic acids (e.g., Salvianolic acid B), alkaloids (e.g., Sinomenine), coumarins (e.g., Osthole), diterpenoids (e.g., Triptolide) and triterpenoids (e.g., Celastrol, Betulinic acid), has been proved in a CIA model [17,18]. These bioactive compounds ameliorate arthritic symptoms through multiple and different targets involved in the inflammatory response, immunoregulation, oxidative stress, angiogenesis and miRNA [17]. In order to show this, Sinomenine demonstrated an anti-arthritic effect by inhibiting the production of pro-inflammatory factors and the monocyte/macrophages population [19], and angiogenesis [20]. The anti-arthritic effect of polyphenols (flavonoids and phenolic acids) is mainly due to their anti-inflammatory, antioxidant and proapoptotic mechanisms [21]. Apart from single phytoconstituents, some plant extracts such as the water extract of Acori Graminei Rhizoma and the ethyl acetate extract of the traditional Tibetan medicine Rhamnella gilgitica have been shown to have a significant anti-arthritis effect in the CIA model by inhibiting pro-inflammatory cytokines [22,23].

The genus *Teucrium* L. comprises about 200 species spread throughout all continents, especially in the Mediterranean region. *Teucrium montanum* L., or mountain germander (TM), is a perennial shrub native to Europe, Northern Africa and Turkey. The *Teucrium* species contain different phenolic acids (gallic, chlorogenic, caffeic and ferulic acid), flavonoids (rutin and luteolin derivatives), phenylethanoids (verbascoside), bitter compounds and essential oils (germacrene D, α-pinene, β-eudesmol and β-caryophyllene) [24,25,26]. *Teucrium montanum* L. has been used in traditional medicine for centuries for the treatment of various kinds of ailments and disorders, including diabetes, cardiovascular disease, abdominal pain, intestinal spasms, diarrhea, liver damage, tuberculosis, rheumatism, etc. [24,25,26]. *Teucrium montanum* L. is thought to strengthen the immune system [27], although data on the effects of TM on the cells of the immune system are very limited. A recent study reported that the dichloromethane TM extract stimulates lymphocyte proliferation and modulates the differentiation of T cells in vitro [28]. *Teucrium montanum* L. has also been suggested to be able to protect against microbial infection and cancer, especially in the respiratory and digestive tracts [29,30]. Previous studies analyzing the biological activity of TM extracts in vitro have shown that they have antimicrobial, antioxidant, anti-inflammatory, antiproliferative and proapoptotic activity [26,30,31,32], which can be linked to their wide application in traditional medicine. However, caution should be applied with excessive consumption due to the previously recognized hepatotoxicity of some *Teucrium* species, such as *T. chamaedrys* and *T. polium* [33,34]. To date, to our best knowledge, TM extracts have not been investigated in experimental models or in clinical studies.

Currently, CIA, the most frequently used in vivo model of RA, represents the gold standard and important tool in investigation of new therapies for RA [35]. This model mimics human disease in various clinical and pathogenic aspects of the disease [35,36,37,38,39]. Our preliminary experiments showed favorable effect of TM ethanol extract on clinical manifestation of arthritis in *Dark Agouti* (DA) rat CIA model. Thus, this study aimed to evaluate, for the first time, therapeutic effects of TM extract on CIA, with focus on elucidating its effects on main cellular and molecular components of innate and adaptive immunity that have a role in pathogenesis of RA and CIA.

## 2. Materials and Methods

### 2.1. Plant Material and Extraction

The aerial parts of TM in the flowering stage were collected from Vratna gorge in eastern Serbia in July 2020. The plant material was dried at room temperature (RT). The plant material was extracted with ethanol (1:10; ethanol 96% *v*/*v*) for 24 h at room temperature to ensure the stability of potentially thermolabile compounds. Low-toxicity ethanol can dissolve polar constituents (phenolic acids, flavonoids, phenylethanoid glycosides) and non-polar constituents (essential oils). The extraction was repeated due to the exhaustion of the material, the extracts were united, and the solvent evaporated under the reduced pressure.

### 2.2. LC-MS Analysis

The LC-MS analysis was performed on an Agilent LC/MS system 1260/6130 (Agilent Technologies, Waldbronn, Germany) using a Zorbax SB-Aq column (150 × 3.0 mm; 3.5 μm particle size, Agilent Technologies) according to [40]. The identification and quantification were performed via the external standard method. The contents were calculated based on the peak areas obtained by DAD (at 320 or 350 nm) using the calibration curves obtained for seven commercial standards. The regression equations, correlation coefficients (r^2^), linear ranges, limits of detection (LODs) and limits of quantification (LOQs) in μg/mL are given in Table S1. The LODs and LOQs were calculated using a signal-to-noise ratio according to the guidelines of the International Conference on Harmonization [41].

### 2.3. Experimental Animals

Female four-month-old DA rats (180 g–210 g), bred at the Institute of Virology, Vaccines and Sera “Torlak” animal facility (Belgrade, Serbia) and authorized for use by The Ministry of Agriculture, Forestry and Water Economy of the Republic of Serbia, were used in this study. The rats were kept in polyethylene cages in an environment under standardized conditions, i.e., 12 h light/dark cycle, 22  ±  1 °C temperature, and 55  ±  5% humidity. A standard food diet for rats was used for feeding and access to tap water was ad libitum. Daily inspections of the healthiness of the rats were carried out by a veterinarian and trained animal care staff. The design and methods applied were performed in keeping with Directive 2010/63/EU of the European Parliament and the Council for the protection of animals used for scientific purposes and governmental regulations (The Law on Animal Welfare, “Official Gazette of Republic of Serbia”, no. 41/2009), and were approved by The Ministry of Agriculture, Forestry and Water Economy of the Republic of Serbia (permit no. 323-07-12928/2022-05/02). The ARRIVE guidelines for reporting animal research were fully implemented in this study.

### 2.4. Induction and Clinical Assessment of CIA

Immunization for the induction of CIA was performed intradermally at the tail base of the rats with an emulsion that contained 300 μg of bovine collagen type II (CII) (Sigma-Aldrich Chemie GmbH, Taufirchen, Germany) in incomplete Freund’s adjuvant (IFA) [36]. The rats were previously anesthetized with an intraperitoneal injection of ketamine/xylazine cocktail (50 mg/kg body weight (BW) of ketamine/5 mg/kg BW xylazine). The development of arthritis was monitored on the paws of all 4 limbs daily. The signs of arthritis, namely the swelling and redness of joints, were scored as following: 1 point for each inflamed metacarpophalangeal/metatarsophalangeal joint or proximal interphalangeal joint of each toe, and 5 points for an inflamed wrist/ankle joint. Thus, the maximal individual score for each paw was 15, and the highest arthritis score was 60, as previously suggested [42]. The evaluation of disease development was performed by experienced researchers who were blinded to the experimental design.

### 2.5. Experimental Design

At 13 d.p.i, when the first clinical signs of arthritis appeared, the rats were randomly assigned to groups (6 per group): a group treated with a dose of 100 mg/kg (TM100) (CIA + TM100), a group treated with a dose of 200 mg/kg (TM200) (CIA + TM200), and a group who received vehicle (saline) (CIA). The TM extract was administered daily by oral gavage from the 13th day until the 22nd day after immunization, following the guidelines on refinement in RA research [43]. The doses were chosen based on data available in the literature regarding the anti-inflammatory doses of other *Teucrium* species used in vivo [44], and from our preliminary experiment that showed that the TM extract at these doses reduces the clinical signs of CIA in rats. Healthy rats were administered with saline in the same way (healthy controls, 6 rats per group).

On the 22nd d.p.i., the animals were deeply anesthetized with ketamine (80 mg/kg BW/xylazine/8 mg/kg BW), and their blood, dLNs (popliteal), spleens, livers and hind paws were taken for further analyses.

### 2.6. Serum Collection

Blood samples, taken from the heart of the animals, were left to coagulate for one hour at RT and then centrifuged at 2000× *g* for 10 min to collect sera. The sera were decomplemented by incubation for 30 min at 56 °C and stored at −20 °C until further analyses.

### 2.7. Cell Preparation

The preparation of single-cell suspensions was performed by passing the dLNs and spleens through a 70 μm nylon cell strainer (BD Biosciences, Erembodegem, Belgium) in Petri dishes with ice-cold phosphate-buffered saline (PBS) supplemented with 2% fetal calf serum (FCS, Gibco, Grand Island, NY, USA). An improved Neubauer hemocytometer and trypan blue dye were used to count viable cells and exclude non-viable cells. The cells were subjected to immunolabelling and cultivation.

### 2.8. Paw Culture and Secreted Cytokines Determination

Hind paws were collected by making an incision at the fur line and processed for cultivation as previously described [37]. Briefly, the paws were minced up into small pieces and cultured in RPMI 1640 medium (Sigma-Aldrich Chemie GmbH) supplemented with 2 mM of l-glutamine (Serva, Heidelberg, Germany), 1 mM of sodium pyruvate (Serva), 100 units/mL penicillin (ICN, Costa Mesa, CA, USA), 100 μg/mL streptomycin (ICN) and 10% FCS overnight (37 °C, humidified air atmosphere of 5% *v*/*v* CO_2_). Supernatants were collected for TNF-α, IL-10, and IL-6 ELISA assay determination.

For measuring the concentrations of TNF-α (BioLegend, San Diego, CA, USA; detection limit 2 pg/mL), IL-10 (R&D Systems, Minneapolis, MN, USA; detection limit 10 pg/mL) and IL-6 (BioLegend; detection limit 5.3 pg/mL) in the supernatants of the paw tissue cultures, commercial ELISA kits were used. All procedures were performed according to the manufacturers’ instructions.

### 2.9. Histopathological Analysis

For the histopathological analyses, left legs were immediately fixed in 10% neutral-buffered formalin and then subjected to decalcification in decalcifying solution [8% HCl from 37% (*v*/*v*) concentrate and 10% formic acid from 89% (*v*/*v*) concentrate in PBS] for approximately 24 h at 37 °C. Following decalcification, the tissues were embedded in paraffin blocks. Livers were also fixed in 10% neutral-buffered formalin for 48 h at RT, routinely processed and embedded in paraffin blocks. Then, 5 μm thick sections were stained with hematoxylin and eosin (H&E) to reveal the tissue structures and analyzed by light microscopy (Olympus BX 41) by pathologists who were blinded to the experimental conditions.

### 2.10. Cell Immunostaining and Flow Cytometry Analysis

For immunolabeling, the following monoclonal antibodies (mAbs) were used:

FITC-conjugated anti-rat CD8 (clone OX-8), PE-conjugated anti-rat CD4 (clone OX-38), PerCP-conjugated anti-rat TCRαβ (clone R73), FITC-conjugated anti-IFN-γ (clone DB-1), PE-conjugated anti-IL-17A (clone TC11-18H10), and PE-Cy 5-conjugated anti-rat CD45RA (clone OX-33) were obtained from BD Biosciences Pharmingen (Mountain View, CA, USA), and biotin-conjugated anti-CD86 (clone 24F), PE-conjugated anti-MHC class II (clone OX-6), FITC-conjugated anti-mouse/rat FoxP3 (clone FJK-16s), and allophycocyanin-conjugated anti-CD4 (clone OX35) were obtained from eBioscience (San Diego, CA USA). Also, FITC-conjugated anti-rat CD11b (clone ED8) (Bio-Rad, Hercules, CA, USA), eFluor 660-conjugated anti-rat TNF-α (clone TN3-19.12) (Invitrogen, Carlsbad, CA, USA), polyclonal rabbit anti-TLR4 antibody (Abcam, Cambridge, UK), and polyclonal rabbit anti-rat IL-1β (Novus Biologicals, Littleton, CO, USA) were used. Streptavidin-PerCP (BioLegend), PE-conjugated donkey F(ab’)2 and anti-rabbit IgG H&L (Abcam) were used as second-step reagents.

For surface immunolabeling, the dLN and spleen cells were incubated with saturating concentrations of either fluorochrome-conjugated or unconjugated/biotin-conjugated monoclonal antibodies, followed by incubation with a second-step reagent.

For intracellular cytokine staining, freshly isolated dLN and spleen cells were firstly cultivated in 24-well plates in 500 μL of culture medium with 200 ng/mL phorbol 12-myristate 13-acetate (PMA, Sigma-Aldrich Chemie GmbH), 400 ng/mL of ionomycin (Sigma-Aldrich Chemie GmbH) and 3 µg/mL of brefeldin A (eBioscience) for 4 h at 37 °C and in a humidified air atmosphere of 5% *v*/*v* CO_2_), harvested and subjected to immunolabeling.

Intracellular staining for cytokine expression and FoxP3 expression in freshly isolated dLN cells and splenocytes was performed following surface immunostaining and overnight fixation/permeabilization with reagents from eBioscience, in compliance with the manufacturer’s instructions. Between steps, the cells were washed with permeabilization buffer (eBioscience).

All data were acquired on a FACSCalibur flow cytometer (Becton Dickinson, Mountain View, CA, USA) and analyzed by using FlowJo software version 7.8. (TreeStar Inc., Ashland, OR, USA) for the frequency of positive marker cells, and the changes in the mean fluorescence intensity (MFI). Gating boundaries were set up using IgG isotype- and fluorochrome-matched and fluorescence minus one (FMO) controls.

### 2.11. Anti-CII Antibody ELISA

The serum levels of anti-CII IgG antibodies were detected by ELISA as described earlier [36]. Briefly, wells of 96-well plates (MaxiSorp, Nunc, BioLegend) were coated with 5 μg/mL of CII (50 μL/well) in 50 mM of carbonate buffer at pH 9.6 by overnight adsorption at +4 °C and blocked with 2% BSA in PBS for 1 h at RT. Dilutions of sera (1:100) were added to the wells (50 μL/well). After overnight incubation at 4 °C and a washing step (0.05% Tween 20/PBS; 4 × 200 μL/well), biotin-conjugated secondary antibody anti-rat IgG (1:1000) (Biolegend Inc., San Diego, CA, USA) was added for another incubation (1 h at the RT). Following incubation and the washing step, the ExtrAvidin-peroxidas e/o-phenylendiamine system (Sigma, Steinheim, Germany) was used for the detection of specific antibodies. The reaction was stopped by an addition of 1 M H_2_SO_4_ (50 μL/well) and the absorbance was read at 492/620 nm (A492/620) on Multiscan Ascent (Labsystems, Helsinki, Finland).

### 2.12. Oxidative Stress Analysis

The sera obtained on the 22nd d.p.i. were tested for the levels of oxidative stress parameters. The total oxidant capacity (TOC), total antioxidant capacity (TAC), and prooxidant-antioxidant balance (PAB) were assessed by modified spectrophotometric methods using o-dianisidine [45], ABTS [46], and 3, 3′, 5, 5′-tetramethylbenzidine [47] as a chromogen [48], respectively. The number of sulfhydryl groups (SHGs) was measured using dinitrodithiobenzoic acid as a reagent [48]. The number of advanced oxidation protein products (AOPPs) was assessed using a reaction with glacial acetic acid and potassium iodide [49]. Superoxide dismutase (SOD) activity was measured according to the method of Misra and Fridovich [50]. This method is based on the SOD-mediated inhibition of adrenalin autooxidation to adrenochrome. One unit of SOD activity is defined as the activity that inhibits adrenalin auto-oxidation by 50%, and the results are expressed as units per liter (U/L). Spectrophotometric assays were performed with a continuous spectrophotometer (Pharmacia LKB, Cambridge, UK), except for assays for the determination of TAC and TOC, which were performed on a ILAB 300+ analyzer (Instrumentation Laboratory, Milan, Italy). Chemicals were purchased from Sigma-Aldrich Chemie (Munich, Germany).

### 2.13. Aspartate Aminotransferase and Alanine Aminotransferase Determination

The serum levels of aspartate aminotransferase (AST) and alanine aminotransferase (ALT) were assayed by routine enzymatic methods using the ILAB 300+ analyzer and the reagents purchased from Biosystems (Biosystems, Barcelona, Spain).

### 2.14. Statistical Analysis

Data were analyzed using GraphPad Prism 6 software (GraphPad Software, Inc., La Jolla, CA, USA). Statistically significant differences between the groups were assessed by the Kruskal–Wallis test, followed by the Mann–Whitney U test. Data are presented as median with interquartile range. Values of *p* ≤ 0.05 were considered significant.

## 3. Results

### 3.1. Composition of T. montanum Extract

In the ethanol extract of TM aerial flowering parts, seven phenolic compounds were identified using LC-MS (the spectral data and results of the quantitative analysis are given in Table 1). The quantitative analysis of the extract was performed by the external standard method using the peak areas obtained by DAD (at 320 and 350 nm). The most abundant was phenylethanoid glycoside verbascoside (6.1%), followed by flavonoid luteolin 7-*O*-rutinoside (0.9%).

### 3.2. Extract T. montanum Improved Clinical and Histopathological Signs of Arthritis in CIA Rats

**Figure 1:**
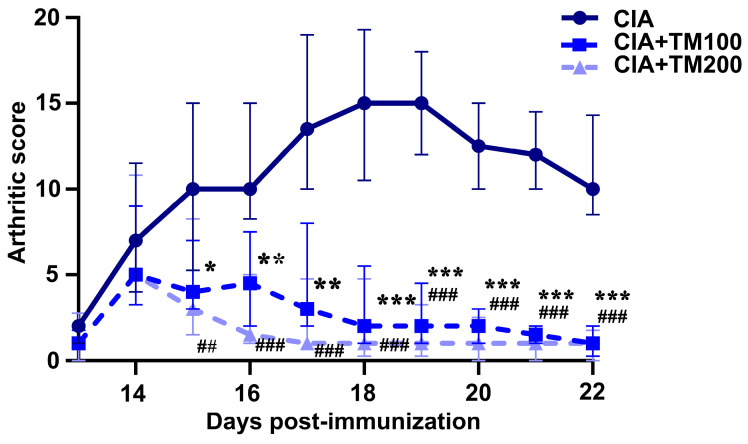
The effect of *T. montanum* ethanol extract treatment on the clinical course of CIA in rats. Daily arthritic score (median with interquartile range) in CIA rats and CIA rats treated with 100 mg/kg of *T. montanum* extract (TM) (CIA + TM100) and 200 mg/kg of TM (CIA + TM200) from the 13th day until the 22nd day post-immunization, with bovine collagen type II in incomplete Freund’s adjuvant. Clinical signs (joint swelling and redness) of arthritis were graded on an arbitrary scale as follows: 1 point for each inflamed metacarpophalangeal/metatarsophalangeal joint or proximal interphalangeal joint of each toe, and 5 points for an inflamed wrist/ankle joint. Thus, the maximal individual score for each paw was 15, and the highest arthritis score was 60. * *p* ≤ 0.05, ** *p* ≤ 0.01, *** *p* ≤ 0.001 for CIA vs. CIA + TM100; ## *p* ≤ 0.01, ### *p* ≤ 0.001 for CIA vs. CIA + TM200; *n* = 6 rats/group.

Upon the appearance of the first clinical signs of arthritis, CIA rats were treated with the TM extract at doses of 100 mg/kg or 200 mg/kg via oral administration once a day. Regardless of the administered dose, the TM extract significantly attenuated the clinical signs of arthritis, as indicated by a significant reduction in the arthritis score (Figure 1). The TM extract led to an improvement in the clinical signs of arthritis after two days of treatment, and this improvement was maintained until the end of the experiment (Figure 1).

**Figure 2:**
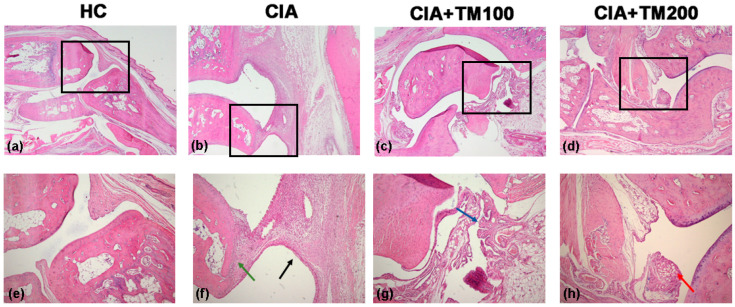
Histopathological analysis of ankle joints of CIA rats treated with *T. montanum* extract. Representative microphotographs from H&E-stained sections of ankle joints of CIA rats, CIA rats treated with 100 mg/kg of *T. montanum* extract (TM) (CIA + TM100) and 200 mg/kg of TM (CIA + TM200), and healthy control rats (HC). (**a**,**e**) HC: no evidence of inflammation, no cartilage destruction, regular subchondral bone; (**b**,**f**) CIA: intensive diffuse mononuclear infiltrates, hyperplasia of the synovial layer with superficial necrosis (black arrow), and cartilage and bone destruction (green arrow); (**c**,**g**) CIA + TM100: reduction in inflammation, focal mononuclear infiltrate, no necrosis, papillary synovial appearance (blue arrow), and only cartilage destruction; (**d**,**h**) CIA + TM200: no inflammation, no necrosis, focally synovial fatty metaplasia and fibrosis (red arrow), and minimal cartilage destruction. Magnification: 100× and 400×.

The histopathological analysis of tibiotarsal (ankle) joints in CIA rats showed intensive diffuse mononuclear cell infiltration, synovial hyperplasia with superficial necrosis, and cartilage and bone destruction (Figure 2). The TM extract-treated CIA rats showed a reduction in inflammation, mononuclear cell infiltration and synovial layer superficial necrosis in the ankle joints. The attenuating effects of the TM extract on the histological features of ankle joints in CIA rats were more pronounced at a higher applied dose (Figure 2).

Based on data in the literature [33,34] showing that some *Teucrium* species, such as *T. chamaedrys* and *T. polium* have a hepatotoxic effect, we measured AST and ALT, hepatic enzyme markers of liver damage, in the sera of CIA and TM extract-treated CIA rats. The levels of AST [222 (64.7) IU/L in CIA, 189.9 (37.7) IU/L in CIA + TM100, 148.7 (40.9) IU/L in CIA + TM200] and ALT [56.10 (6.53) IU/L in CIA, 38.5 (2.6) IU/L in CIA + TM100, 46.40 (9.9) IU/L in CIA + TM200] did not differ statistically between the CIA and TM-extract treated groups. Additionally, the histopathological analysis of the liver tissue did not show differences between the CIA and TM extract-treated groups.

### 3.3. T. montanum Extract Promoted Anti-Oxidant Activity in CIA Rats

**Figure 3:**
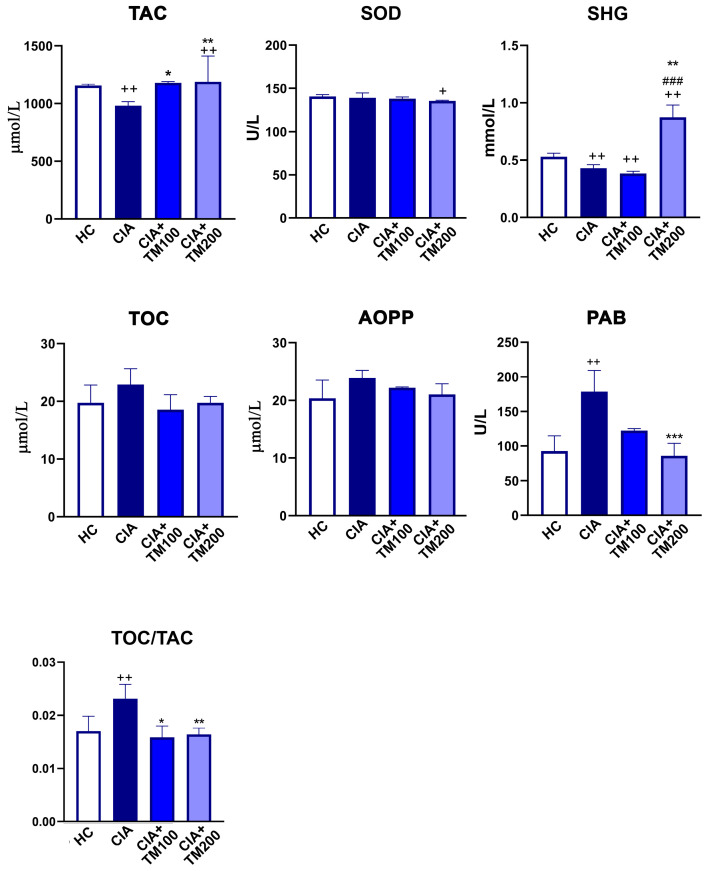
The effect of *T. montanum* extract on the oxidative stress parameter levels in the serum of CIA rats. The bar graphs represent the levels of antioxidative parameters: total antioxidant capacity (TAC), superoxide dismutase (SOD), sulfhydryl groups (SHG); prooxidative parameters: total oxidant capacity (TOC), advanced oxidation protein products (AOPPs), pro-oxidant–antioxidant balance (PAB), and TOC/TAC ratio. These parameters were determined in the sera of CIA rats, CIA rats treated with 100 mg/kg of *T. montanum* extract (TM) (CIA + TM100) and 200 mg/kg of TM (CIA + TM200), and healthy control rats (HC). Results are expressed as median with interquartile range. * *p* ≤ 0.05, ** *p* ≤ 0.01, and *** *p* ≤ 0.001 for TM-treated CIA vs. CIA; ### *p* ≤ 0.001 for CIA + TM100 vs. CIA + TM200; + *p* ≤ 0.05, ++ *p* ≤ 0.01 vs. HC. *n* = 6 rats/group.

Considering that oxidative-stress-related parameters are considered as potential biomarkers of RA activity [51], and that plant extracts are known to be rich in compounds with antioxidant activity [52], the redox status of the serum from CIA rats and TM extract-treated CIA rats was examined. The values of the anti-oxidative parameters TAC and SHG were higher, whereas SOD remained unaltered in the TM extract-treated CIA rats compared with CIA rats (Figure 3). The pro-oxidative parameter PAB was lower in the TM-treated CIA rats than in the CIA rats, while TOC and AOPP did not differ between these experimental groups (Figure 3). The oxidant-to-antioxidant balance, estimated using the TOC/TAC ratio, was lower in TM-treated CIA rats compared with CIA rats (Figure 3).

### 3.4. T. montanum Extract Reduced Production of Pro-Inflammatory Cytokines While Increased Production of Anti-Inflammatory Cytokines in Cia-Rats

**Figure 4:**
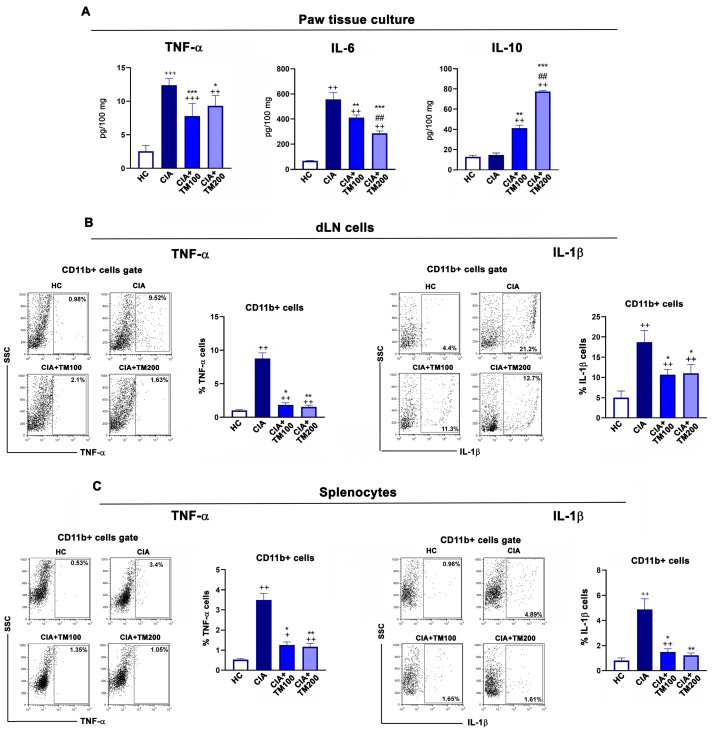
The effect of *T. montanum* extract on pro- and anti-inflammatory cytokine production in the arthritic paws, draining lymph nodes and spleens of CIA rats. (**A**) The bar graphs represent the concentrations of TNF-α, IL-6 and IL-10 in the supernatants from the hind paw tissue cultures (normalized to the paw weight) of CIA rats, CIA rats treated with 100 mg/kg of T. montanum extract (TM) (CIA + TM100) and 200 mg/kg of TM (CIA + TM200), and healthy control rats (HC). Results are expressed as median with interquartile range. (**B**,**C**) The bar graphs represent the percentage of TNF-α+ and IL-1β+ cells within the CD11b+ cells of (**B**) draining lymph node (dLN) cells and the (**C**) splenocytes of CIA rats, CIA + TM100, CIA + TM200 and HC. Results are expressed as median with interquartile range. Representative flow cytometry dot plots indicate the percentage of TNF-α+ cells and IL-1β+ cells among CD11b+ cells from the (**B**) dLNs and (**C**) spleens gated, as shown in Figure S1. * *p* ≤ 0.05, ** *p* ≤ 0.01, and *** *p* ≤ 0.001 for TM-treated CIA vs. CIA; ## *p* ≤ 0.01 for CIA + TM100 vs. CIA + TM200; + *p* ≤ 0.05, ++ *p* ≤ 0.01, and +++ *p* ≤ 0.001 vs. HC. *n* = 6 rats/group.

Pro-inflammatory and anti-inflammatory cytokines play a critical role in the pathogenesis of RA [53] and CIA [35], and therefore we determined the levels of TNF-α, IL-6 and IL-10 in paw tissue cultures. The concentrations of the pro-inflammatory cytokines TNF-α and IL-6 were lower, while that of anti-inflammatory IL-10 was higher in the paw tissue cultures from TM-treated CIA rats compared with CIA rats (Figure 4A). Consistent with this, compared with CIA rats, in the dLNs and spleens from TM-treated CIA rats, lower percentages of CD11b+ cells expressing the pro-inflammatory cytokines TNF-α and IL-1β were found (Figure 4B,C).

### 3.5. T. montanum Extract Affected Activation Status of Antigen-Presenting Cells in Secondary Lymphoid Organs

**Figure 5:**
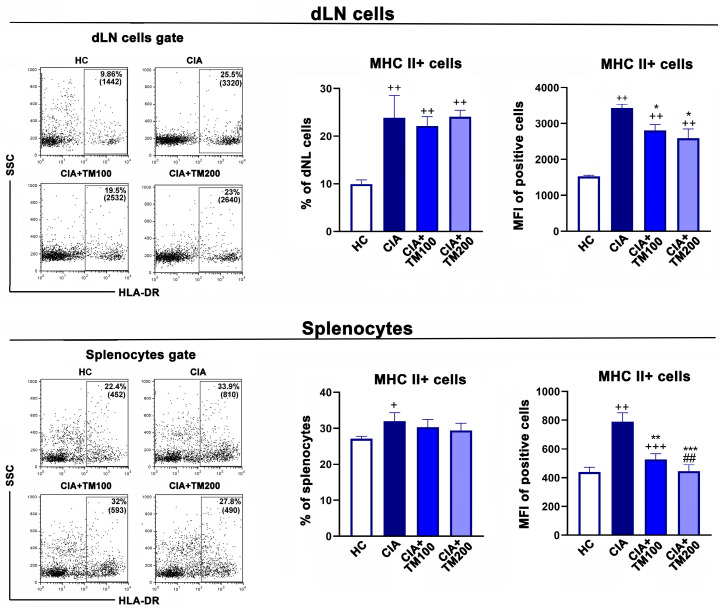
The effect of *T. montanum* extract on the expression of MHC II molecules in cells from the draining lymph nodes and spleens of CIA rats. The bar graphs represent the percentages of MHC II+ cells and the mean fluorescent intensity (MFI) of MHC II+ cells within (upper row) the draining lymph node (dLN) cells and (lower row) splenocytes of CIA rats, CIA rats treated with 100 mg/kg of T. montanum extract (TM) (CIA + TM100) and 200 mg/kg of TM (CIA + TM200), and healthy control rats (HC). Results are expressed as median with interquartile range. Representative flow cytometry dot plots indicate the percentage of MHC II+ cells and the MFI (in parenthesis) of MHC II+ cells of the dLN cells and splenocytes gated, as shown in the FSC/SSC dot plots in Figure S1A. * *p* ≤ 0.05, ** *p* ≤ 0.01, and *** *p* ≤ 0.001 for TM-treated CIA vs. CIA; ## *p* ≤ 0.01 for CIA + TM100 vs. CIA + TM200; + *p* ≤ 0.05, ++ *p* ≤ 0.01, and +++ *p* ≤ 0.001 vs. HC. *n* = 6 rats/group.

The activation status of antigen-presenting cells has an important role in the pathogenesis of CIA and RA, and can influence both the development and severity of the disease [35,53,54,55,56]. Therefore, we examined MHCII and CD86 expression in the dLNs and spleens from CIA and TM-treated CIA rats. Compared with CIA rats, the MFI of the MHC II molecules was diminished in cells retrieved from the dLNs and spleens of TM-treated rats (Figure 5).

**Figure 6:**
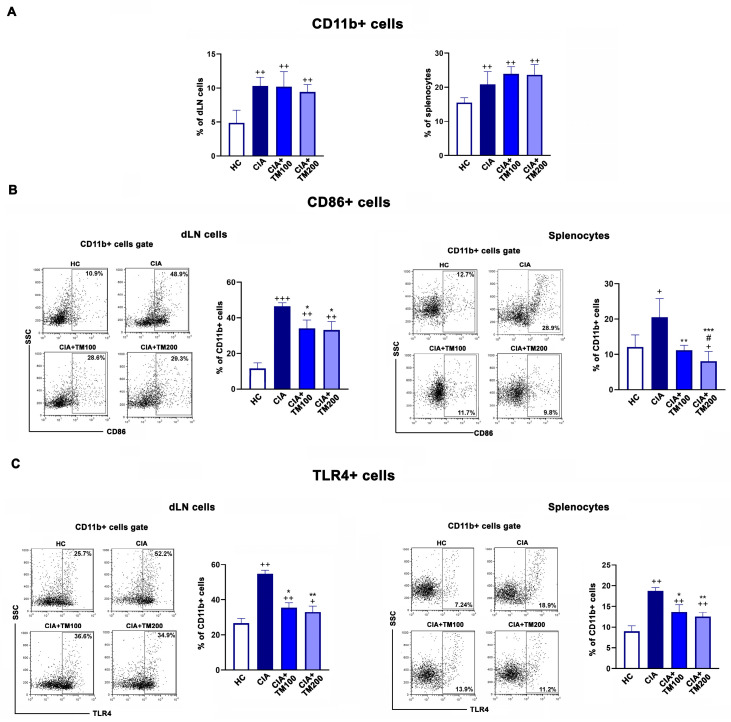
The effect of *T. montanum* extract on CD86 and TLR4 expression in cells from the draining lymph nodes and spleens of CIA rats. (**A**) The bar graphs represent the percentage of CD11b+ cells within the draining lymph node (dLN) cells and splenocytes of CIA rats, CIA rats treated with 100 mg/kg of T. montanum extract (TM) (CIA + TM100) and 200 mg/kg of TM (CIA + TM200), and healthy control rats (HC). Results are expressed as median with interquartile range. The gating strategy for the flow cytometry analysis of the CD11b staining of dLN cells and splenocytes is presented in Figure S1A. The bar graphs represent the percentage of (**B**) CD86+ and (**C**) TLR4+cells within the dLN cells and splenocytes of CIA rats, CIA + TM100, CIA + TM200, and HC. Results are expressed as median with interquartile range. Representative flow cytometry dot plots indicate the percentage of (**B**) CD86+ cells and (**C**) TLR4+ cells among CD11b+ cells from the dLN cells or splenocytes gated, as shown in Figure S1A. * *p* ≤ 0.05, ** *p* ≤ 0.01, and *** *p* ≤ 0.001 for TM-treated CIA vs. CIA; # *p* ≤ 0.05 for CIA + TM100 vs. CIA + TM200; + *p* ≤ 0.05, ++ *p* ≤ 0.01, and +++ *p* ≤ 0.001 vs. HC. *n* = 6 rats/group.

Further, the frequency of CD11b+ cells, presumably antigen-presenting cells, in the dLNs and spleens was similar in the examined groups, but compared with CIA rats, a lower percentage of costimulatory molecule CD86+ cells among CD11b+ cells was found in the TM-treated CIA rats (Figure 6A,B). Moreover, there are data indicating that Toll-like receptors (TLRs), especially TLR4, play important pathogenic roles in RA and CIA [57,58,59]. We found lower percentages of TLR4+ cells among CD11b+ cells from both the dLNs and spleens of TM-treated CIA rats compared with CIA rats (Figure 6C).

### 3.6. T. montanum Extract Affected T-Cell Mediated Response in CIA Rats

**Figure 7:**
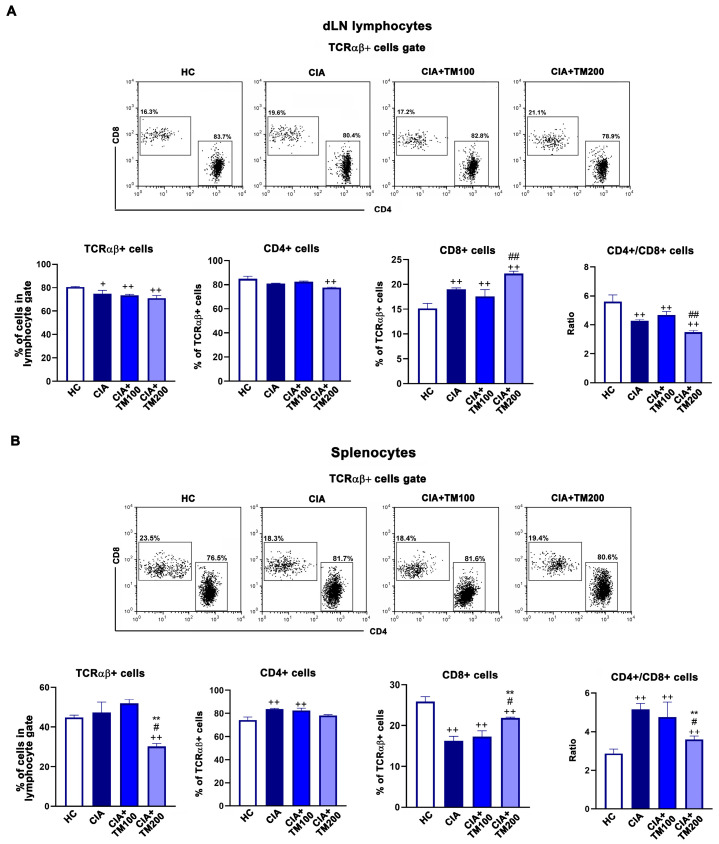
The effect of *T. montanum* extract on the frequency of TCRαβ+ cells and CD4+ and CD8+ subpopulations in the draining lymph nodes and spleens of CIA rats. The bar graphs represent the percentages of TCRαβ+ cells and CD4+ and CD8+ cells among the TCRαβ+ cell population and CD4+/CD8+ cell ratio in the lymphocytes of the (**A**) draining lymph nodes (dLN) and (**B**) spleens of CIA rats, CIA rats treated with 100 mg/kg of T. montanum extract (TM) (CIA + TM100) and 200 mg/kg of TM (CIA + TM200), and healthy control rats (HC). Results are expressed as median with interquartile range. Representative flow cytometry dot plots indicate the percentage of CD4+ and CD8+ cells among TCRαβ+ cells from the lymphocyte gate among (**A**) dLN cells and (**B**) splenocytes. The gating strategy for the flow cytometry analysis of TCRαβ+ cells from the lymphocyte gate is presented in Figure S1B. ** *p* ≤ 0.01 for TM-treated CIA vs. CIA; # *p* ≤ 0.05, and ## *p* ≤ 0.01 for CIA + TM100 vs. CIA + TM200; + *p* ≤ 0.05, and ++ *p* ≤ 0.01 vs. HC. *n* = 6 rats/group.

It is a longstanding belief that RA is a T cell-mediated autoimmune disease, with both T cell subsets and CD4+ and CD8+ cells having a role in its pathogenesis [60]. Several studies point to a greater CD4+/CD8+ T cell ratio in the peripheral blood and synovial fluid of RA patients compared to healthy controls [61,62]. Thus, we assessed the frequency of TCRαβ+, CD4+ and CD8+ T cells and their ratio in the dLNs and spleens of CIA rats and TM-treated CIA rats. The frequency of TCRαβ+ cells and frequency of CD4+ and CD8+ cells among TCRαβ+ dLN cells did not differ between the TM-treated CIA rats and CIA rats (Figure 7A. However, in the spleens of the TM-treated CIA rats, the frequency of TCRαβ+ cells and the CD4+/CD8+ cell ratio among TCRαβ+ cells were lower than in CIA rats (Figure 7B).

**Figure 8:**
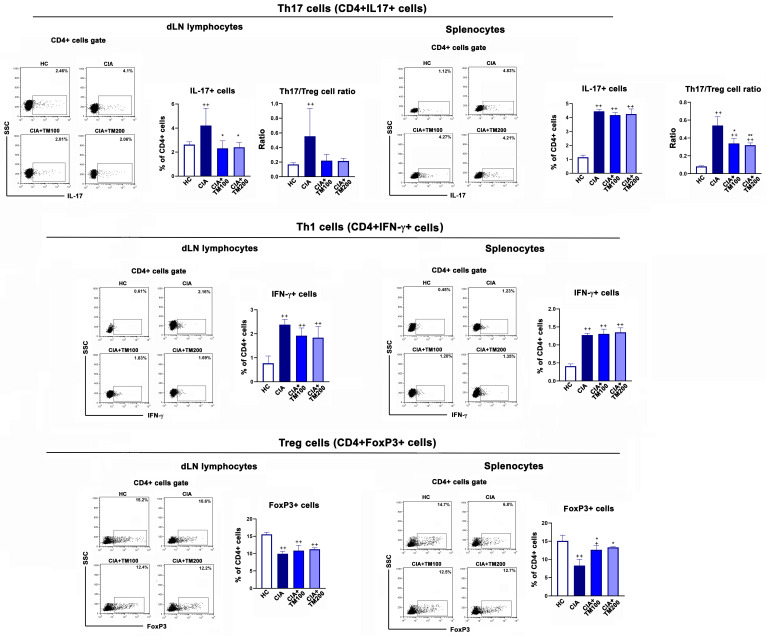
The effect of *T. montanum* extract on frequency of Th17, Th1, and T regulatory cells in the draining lymph nodes and spleens of CIA rats. The bar graphs represent the percentage of IL17+, IFN-γ+ and FoxP3+ cells among CD4+ cells, i.e., Th17, Th1 and T regulatory (Treg) cells, and the Th17/Treg cell ratio in the lymphocytes of the draining lymph nodes (dLN) and spleens of CIA rats, CIA rats treated with 100 mg/kg of T. montanum extract (TM) (CIA + TM100) and 200 mg/kg of TM (CIA + TM200), and healthy control rats (HC). Results are expressed as median with interquartile range. Representative flow cytometry dot plots indicate the percentage of IL-17+ cells, IFN-γ+ cells and FoxP3+ cells among CD4+ cells from the lymphocyte gate among dLN cells and splenocytes, whose gating strategy is shown in Figure S1C. * *p* ≤ 0.05, and ** *p* ≤ 0.01 for TM-treated CIA vs. CIA; + *p* ≤ 0.05 and ++ *p* ≤ 0.01 vs. HC. *n* = 6 rats/group.

Furthermore, CD4+ T cells, i.e., T helper (Th) cells, are a critical population in the pathogenesis of RA, being involved in the induction and propagation of inflammatory responses and the stimulation of the B cell response and autoantibody production [60,63,64]. The Th1 CD4+ T cell subset, whose main cytokine is IFN-γ, and the Th17 CD4+ T cell subset, with IL-17 as the signature cytokine, are the most important Th subsets in the pathogenesis of RA [63,64] and CIA [35]. On the other hand, Tregs have an opposite role to Th17 cells, and are crucial in maintaining self-tolerance and immune homeostasis. Their loss or dysfunction has been confirmed in autoimmune diseases, including RA [65]. An analysis of the expression of IL-17 and IFN-γ among CD4+ cells showed that compared with CIA rats, in the dLNs and spleens of TM-treated CIA rats, the percentage of IL-17+ cells among CD4+ cells was lower, while that of IFN-γ + cells among CD4+ cells remained unaltered. The frequency of Tregs (CD4+FoxP3+ cells) was higher in the spleens of TM-treated CIA rats than those of CIA rats (Figure 8). The Th17/Tregs ratio was lower in both the dLNs and spleens of TM-treated CIA rats compared with CIA rats (Figure 8).

### 3.7. T. montanum Extract Reduced Circulating Level of Anti-CII Antibodies

**Figure 9:**
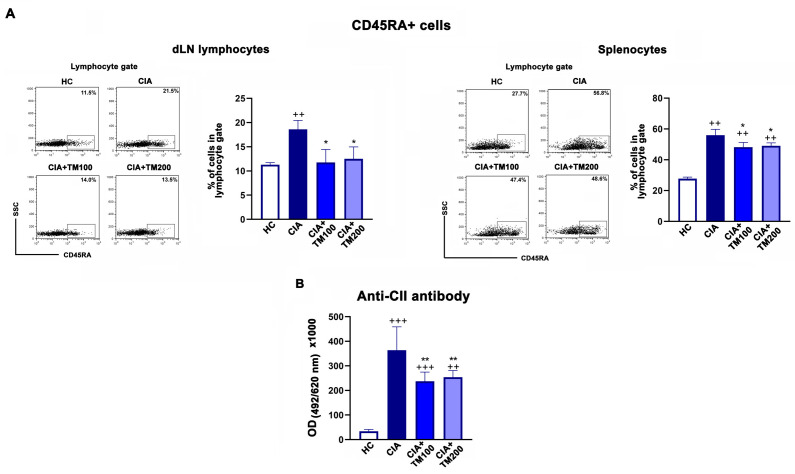
Effect of *T. montanum* extract on the frequency of B cells and the circulating level of anti-CII antibodies. (**A**) The bar graphs represent the percentages of CD45RA+ cells in the lymphocytes of the draining lymph nodes (dLN) and spleens of CIA rats, CIA rats treated with 100 mg/kg of *T. montanum* extract (TM) (CIA + TM100) and 200 mg/kg of TM (CIA + TM200), and healthy control rats (HC). Results are presented as median with interquartile range. Representative flow cytometry dot plots indicate the percentage of CD45RA+ cells in the lymphocyte gate among dLN cells and splenocytes. The lymphocytes were gated as shown in Figure S1B. (**B**) The bar graphs represent the levels [optical density (OD) at 492/620 nm] of anti-CII antibodies in the serum (1:100 dilution) of CIA, CIA + TM100, CIA + TM200, and HC rats. Results are presented as median with interquartile range. * *p* ≤ 0.05, and ** *p* ≤ 0.01. for TM-treated CIA vs. CIA; ++ *p* ≤ 0.01, and +++ *p* ≤ 0.001 vs. HC. *n* = 6 rats/group.

Although T cells play a significant role in the pathogenesis of RA [63,64] and CIA [35], the presence of B cells and their response, which is reflected, among others, in the production of autoantibodies, is important for the development of RA and CIA [11,35]. Irrespective of dose, the TM extract decreased the frequency of B cells (CD45RA+ cells) in the dLNs and spleens of CIA rats (Figure 9). In line with this, the serum levels of IgG anti-CII antibodies, a key pathogenic factor in the CIA model [35], were decreased in TM-treated CIA rats compared with the CIA group (Figure 9). Healthy control rats had negligible serum levels of IgG anti-CII antibodies.

## 4. Discussion

Our study showed for the first time, to the best of our knowledge, that TM extract exerts therapeutic beneficial effects that result in attenuated clinical signs of arthritis and a histological improvement in a CIA rat model. This could be associated with the anti-inflammatory/immunomodulatory properties of TM, which include the suppression of T cell responses in secondary lymphoid organs, decreased pro-inflammatory cytokine production in affected joins, and an improved anti-/pro-oxidative balance in the serum. Furthermore, TM reduced the circulating level of anti-CII antibodies in the treated CIA rats.

Chemical composition varies greatly among *Teucrium* species, but it has been shown that TM has high content of flavonoids and phenolic compounds [66], higher than other *Teucrium* species [25]. Using qualitative and quantitative LC-MS analysis, we identified seven compounds in the TM extract, of which phenylethanoid glycoside verbascoside was the most abundant constituent, followed by flavonoid luteolin 7-*O*-rutinoside. This was generally consistent with previous studies investigating the content of different water, acetone (60%, *v*/*v*), methanol, and ethanol (80%, *v*/*v*) extracts of TM [26,67,68]. Verbascoside is a compound widespread in many plants, and its antioxidant, anti-inflammatory, anti-neoplastic, and wound-healing properties have been shown in in vitro and in vivo models [69,70,71,72,73,74]. Even more, previous studies showed that verbascoside inhibits the expression and DNA binding of the pro-inflammatory transcriptional factors AP-1 and NFκB [73,74,75]. The second most abundant identified constituent of the extract, luteolin 7-*O*-rutinoside, was found to have anti-inflammatory, anti-oxidative and anti-proliferative properties, mostly in in vitro models [76,77].

Oxidative stress, a condition in which the production of reactive oxygen species overcomes the antioxidant defense mechanisms, significantly participates in the pathogenic processes of RA and CIA and is interrelated with other pathogenic mechanisms of the disease [54,78,79,80]. Thus, antioxidant therapy could represent an adjuvant/complementary treatment option [78]. A recent in vitro study reported that TM extracts neutralize 1,1-dyphenyl-2- picrylhydrazyl (DPPH) radicals, suggesting its antioxidant activity [26,66]. The TM extracts obtained with acetone or ethanol solvents had a high content of total polyphenols and total flavonoids and therefore showed better antioxidant activity compared to those obtained using aqueous solvents [26], indicating that the antioxidant capacity of the extract depends on the solvent used for the extraction process. In our study, CIA rats treated with the ethanol TM extract had an improved serum anti-/pro-oxidative balance judging by the upregulated anti-oxidative parameters (TAC and SHG), lowered pro-oxidative PAB parameter, and lowered TOC/TAC score compared to CIA rats, suggesting the antioxidant activity of the extract in vivo. Since oxidative stress has been found to be strongly positively correlated with the inflammatory process and joint destruction in RA patients [78,81], our finding that the TM extract suppresses oxidative stress in CIA rats suggests that anti-oxidative treatment strategies for RA may be effective. To support this, the indicator of the redox status TAC, which has been proposed as a good marker for evaluating RA activity [82,83] since a higher TAC decreases the activity of free radicals, was higher in CIA rats treated with the TM extract than in CIA rats.

Pro-inflammatory cytokines, such as TNF-α, IL-6 and IL-1β, are critical in the propagation of joint inflammation in RA. These cytokines trigger intracellular signaling and lead to the activation of mesenchymal cells and synoviocytes and the recruitment of cells of innate and adaptive immunity into the synovium, starting the vicious cycle that leads further to the production of new amounts of pro-inflammatory cytokines [84]. In line with this, our study showed that the TM extract reduced the production of TNF-α and IL-6 in cultures of the inflamed paws of CIA rats and the frequencies of CD11b+ cells expressing TNF-α and IL-1β in the secondary lymphoid organs of these rats. However, the lesser inflammation and joint tissue damage in TM-treated CIA rats compared with CIA rats could be associated not only with the diminished production of pro-inflammatory cytokines, but also with the increase in anti-inflammatory cytokine IL-10 [85]. IL-10 plays an important role in reducing inflammatory and immune responses, and in allowing tissue repair [86]. Numerous innate and adaptive immune cells, including monocytes/macrophages, Tregs and regulatory B cells, are able to produce IL-10. The major cellular targets of IL-10 are monocytes/macrophages, and many of their pro-inflammatory functions can be inhibited by this cytokine [87]. Thus, it is likely that the ameliorating effect of the TM extract on inflamed joint tissue is not only a consequence of the downregulation of pro-inflammatory cytokines, but also of the upregulation of anti-inflammatory mediators. In line with this, the beneficial effects of the ethanolic extract of *T. polium* in an animal model of acute inflammation [44], and extract of *T. persicum* in animal model of inflammatory bowel disease [88] were accompanied by high expression of IL-10 and low expression of TNF-α and IL-1β.

This study also showed that the TM extract modulated the phenotype characteristics and thus the activity of antigen-presenting cells in CIA rats. Irrespective of the applied dose, the TM extract suppressed the expression of MHC II molecules and the frequency of CD86+ cells among CD11b+ antigen-presenting cells in the dLNs and spleens of CIA rats. MHC II and CD86 molecules have a critical role in the presentation of antigens to CD4+ T lymphocytes and the delivery of the necessary signal for the activation of T cells and the initiation of the T-cell-mediated immune response, respectively [55,56,89]. Therefore, the reduced expression of MHC II and CD86 molecules may suggest the suppression of the T-cell-mediated inflammatory response, which may be one of the mechanisms underlying its therapeutic effect against CIA in rats. In favor of this, in a model of antigen-induced arthritis in mice, the blockade of CD86 reduced the severity of disease, suppressed the production of IL-17 and reduced the accumulation of effector T cells in joints [55].

TLR4 is well known as a molecule that senses pathogens and has a role in protective immunity. However, it has been found that aberrantly expressed or activated TLRs have a role in the pathogenesis of autoimmune diseases [90,91]. Namely, TLR2, TLR3, TLR4 and TLR7 are upregulated in the synovium and synovial macrophages of RA patients [91]. Also, it has been shown that the inhibition of TLR4 in different animal models of arthritis results in improved clinical manifestations and the prevention of the deleterious effects caused by inflammation in joints; this is by suppressing the production of pro-inflammatory cytokines [59,92]. In the present study, lower percentages of TLR4+ cells among CD11b+ cells were found in the dLNs and spleens of TM-treated CIA rats compared with CIA rats, suggesting that the TM extract reduced the expression of TLR4 molecules in antigen-presenting cells. Given that oxidative stress upregulates TLR expression [93], the lower expression of TLR4 could be a consequence of the antioxidant action of the TM extract used for the treatment. It is also possible that the TM extract reduces TLR4 expression through an oxidative stress-independent action since many plant extracts have been shown to modulate the expression and activity of TLR4 receptors [94].

CD4+ T cells are necessary for the induction and development of CIA and for an IgG response towards CII, while CD8+ T cells might have a suppressive role in the etiology of CIA [95]. An increased CD4+/CD8+ T cell ratio due to a decreased frequency of CD8+ T cells has been suggested to indicate RA progression [96]. We found that a higher dose of TM extract significantly decreased the frequency of TCRαβ+ cells and the CD4+/CD8+ T cell ratio (shifted the CD4+/CD8+ T cell ratio towards CD8+ T cells) in the spleens of CIA rats. This suggests that TM may induce a therapeutic effect by lowering the ratio of CD4+/CD8+ T cells.

Furthermore, Th17 cells and Tregs have been suggested to be the major cells regulating the progression of RA [97]. A disturbed peripheral Th17/Tregs balance due to an increase in the frequency of Th17 cells and a decrease in the frequency of Tregs is associated with the development of both RA and CIA [98,99], and the restoration of this imbalance alleviates arthritis and bone destruction [100]. Our results showed that the TM extract restores/improves the Th17/Tregs balance in secondary lymphoid organs. This finding seems significant considering the suggestion that new conventional and traditional drugs for immune bone diseases should have the ability to target and regulate the Th17/Tregs balance [99].

CII-specific antibodies are highly pathogenic in CIA as they form immune complexes with cartilage collagen and activate the complement system and Fc receptor-bearing macrophages to initiate local inflammation [38,39]. We demonstrated herein that the TM extract reduced the serum level of anti-CII antibodies in CIA rats, suggesting that the inhibition of anti-collagen antibody production contributes, at least partly, to the anti-inflammatory effect of the TM extract.

## 5. Conclusions

In conclusion, the TM extract alleviated the clinical manifestation of arthritis in an animal model of RA, which is associated with an improved anti-/pro-oxidative balance, the suppression of inflammatory cytokines and the modulation of the immune response. The use of the extract, which is a mixture of different compounds, is limited by the fact that it is hard to elucidate the influence and contribution of individual components to the overall effect. However, complex mixtures of different compounds, due to the synergistic effects of their components, can have greater effectiveness than drugs based on a single compound, which makes plant extracts more effective in lower doses, and at the same time less toxic. The TM extract improved the clinical symptoms of CIA rats without obvious side effects, indicating that it could potentially be used as a complementary or alternative treatment for RA patients. Further investigations into its mechanisms of action are needed to establish a more solid basis for clinical research.

## Figures and Tables

**Table 1:** The spectral data and the quantities of the identified compounds in *T. montanum* EtOH extract.

No	Rt (min)	*m*/*z* (250 V)	λ_max_, nm	Compound	Content (%, g(100 g)^−1^ dw) *
1	10.43	353 [M − H]^−^, 191, 179, 173	296, 328	Chlorogenic acid	0.317 ± 0.027
2	21.35	623 [M − H]^−^, 461, 315, 161	292, 334	Verbascoside	6.099 ± 0.349
3	21.35	593 [M − H]^−^, 285	254, 268, 350	Luteolin 7-*O*-rutinoside	0.915 ± 0.043
4	22.51	447 [M − H]^−^, 285	254, 268, 350	Luteolin 7-*O*-glucoside	0.344 ± 0.002
5	23.35	577 [M − H]^−^, 269	266, 338	Apigenin 7-*O*-rutinoside	t.r.
6	24.96	431 [M − H]^−^, 269	266, 336	Apigenin 7-*O*-glucoside	0.045 ± 0.003
7	29.49	285 [M − H]^−^, 256, 241, 151, 133	254, 268, 350	Luteolin	0.223 ± 0.032

* The average of three different determinations ± standard deviation; trace-t.r. (<0.001%).

## Data Availability

Data are contained within the article.

## Supplementary Materials

The following supporting information can be downloaded at: https://www.mdpi.com/article/10.3390/biology13100818/s1, Figure S1: Gating strategy for flow cytometric analysis of cells from draining lymph nodes and spleens of CIA rats treated with *T. montanum* extract. (**A**) Representative dot plots indicate gating strategy of (right) CD11b+ cells in (left) cells obtained from draining lymph nodes (dLN) and spleens. (**B**) Representative dot plots indicate gating strategy of (right) TCRαβ+ cells from (left) lymphocyte gate among dLN cells and splenocytes. (**C**) Representative dot plots indicate gating strategy of (right upper row) IL-17+ cells, (right middle row) IFN-γ+ cells and (right lower row) FoxP3+ cells among (left) CD4+ cells from lymphocyte gate indicated in (**B**) among dLN cells and splenocytes; Table S1: Regression equations, correlation coefficients (r^2^), linear ranges, LODs and LOQs of standard substances.
