# Supplementary material for: Evaluation of the Anti-Inflammatory/Immunomodulatory Effect of Teucrium montanum L. Extract in Collagen-Induced Arthritis in Rats
Source: Biology (Basel). 2024 Oct 12;13(10):818. doi: 10.3390/biology13100818 (PMC11505313; doi:10.3390/biology13100818)
Supplement: Supplementary file 1 [file biology-13-00818-s001.zip › biology-3190153-supplementary.pdf]

Supplementary Materials:

The following supporting information can be downloaded at: www.mdpi.com/xxx

Table S1. Regression equations, correlation coefficients (r^2), linear ranges, LODs and LOQs of standard substances.

Regression equation	r^2	Linear range (μg/mL)	LOD (μg/mL)	LOQ (μg/mL)	Compound
y=7359.9706x+21.8506	0.9999	0.05-400	0.015	0.044	Chlorogenic acid
y=3562.6726x+84.8887	0.9998	0.05-500	0.025	0.100	Verbascoside
y=12732.3591x+26.2628	0.9999	0.60-500	0.098	0.250	Luteolin 7-*O*-rutinoside
y=2755.8034x+22.2383	0.9999	0.60-500	0.073	0.238	Luteolin 7-*O*-glucoside
y=4514.5221x–9.4910	0.9995	0.60-400	0.100	0.302	Apigenin 7-*O*-rutinoside
y=29626.4120x+81.6110	0.9994	0.50-500	0.045	0.136	Apigenin 7-O-glucoside
y=2941.0244x+7.5674	0.9999	0.05-3.50	0.030	0.082	Luteolin

LOD – limit of detection; LOQ – limit of quantification

The following supporting information can be downloaded at: www.mdpi.com/xxx/s1, Figure S1: Gating strategy for flow cytometric analysis of CD11b+ cells in draining lymph nodes and spleens of CIA rats treated with *T. montanum* extract**.**

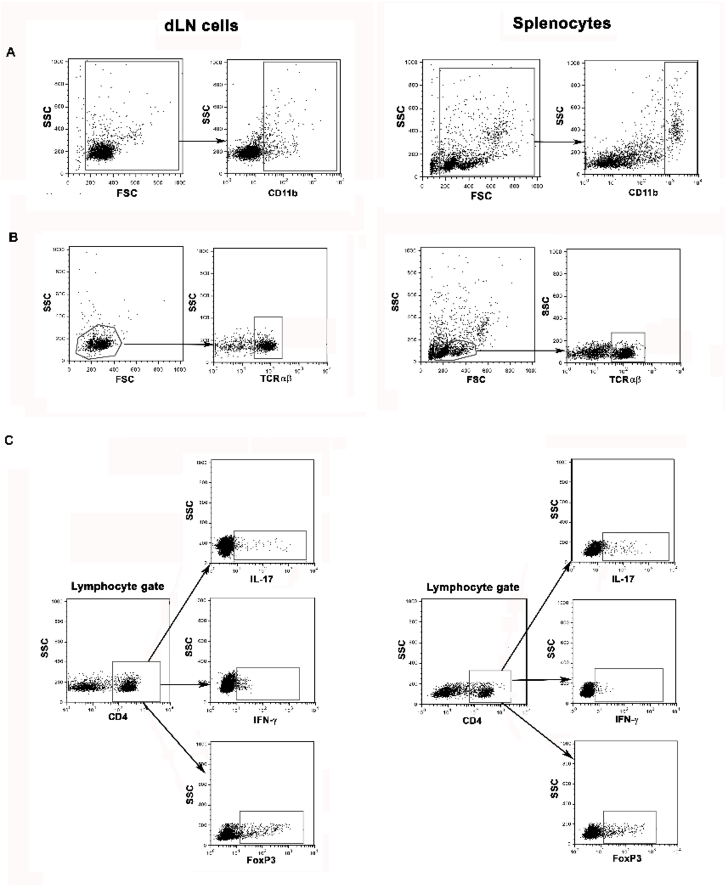

Figure S1. ***Gating strategy for flow cytometric analysis of cells from draining lymph nodes and spleens of CIA rats treated with T. montanum extract.*** (**A**) Representative dot plots indicate gating strategy of (right)

CD11b+ cells in (left) cells obtained from draining lymph nodes (dLN) and spleens. (**B**) Representative dot plots indicate gating strategy of (right) TCRαβ+ cells from (left) lymphocyte gate among dLN cells and splenocytes. (**C**) Representative dot plots indicate gating strategy of (right upper row) IL-17+ cells, (right middle row) IFN-γ+ cells and (right lower row) FoxP3+ cells among (left) CD4+ cells from lymphocyte gate indicated in (**B**) among dLN cells and splenocytes.
